# The Effects of Sodium Selenite on Mitochondrial DNA Copy Number and Reactive Oxygen Species Levels of *In Vitro* Matured Mouse Oocytes

**DOI:** 10.22074/cellj.2018.5430

**Published:** 2018-05-28

**Authors:** Nassim Ghorbanmehr, Mojdeh Salehnia, Mahboobeh Amooshahi

**Affiliations:** 1 Department of Anatomy, Faculty of Medical Sciences, Tarbiat Modares University, Tehran, Iran; 2 Department of Biotechnology, Faculty of Biological Sciences, Alzahra University, Tehran, Iran

**Keywords:** *In Vitro* Maturation, mtDNA, Oocyte, Reactive Oxygen Species, Sodium Selenite

## Abstract

**Objective:**

The aim of present study is to determine the effects of supplementation of oocyte maturation medium with sodium selenite (SS) on oocyte mitochondrial DNA (mtDNA) copy number and reactive oxygen species (ROS) levels.

**Materials and Methods:**

In this experimental study germinal vesicle (GV), metaphase I (MI), and metaphase II (MII) stage oocytes were recovered from 6-8 week old female mice after superovulation. Some of the GV oocytes were cultured and matured in the presence and absence of SS. Then *in vivo* and *in vitro* matured (IVM) oocytes were subjected to mitochondria staining by MitoTracker green, ROS analysis, and mtDNA copy number determination using absolute real-time polymerase chain reaction (PCR).

**Results:**

The maturation rate of GV oocytes to the MII stage significantly increased in the SS supplemented group (79.25%) compared to the control group (72.46%, P<0.05). The intensity of mitochondrial staining was not different among the studied groups, whereas the mitochondria distribution in the cytoplasm of the IVM oocytes showed some aggregation pattern. The *in vivo* obtained MII oocytes had lower ROS levels and higher mtDNA copy numbers than IVM-MII oocytes (P<0.05). The SS supplemented group had significantly lower ROS levels and higher mtDNA copy numbers than the non-treated group (P<0.05).

**Conclusion:**

SS increased oocyte mtDNA copy number by decreasing oxidative stress. SS had an association with better oocyte developmental competence.

## Introduction

Mitochondria are multifunctional organelles with criticalfunctions in ATP production, calcium homeostasis, andcell apoptosis (1). The localization and presence of theseorganelles are critical for successful fertilization (2). Different cell types have a variety of mitochondria that havetheir own genome-mitochondrial DNA (mtDNA) (3). Thereare various reports of mtDNA copy number in oocytes fromdifferent mammalian species (3-7). It is estimated that ahuman MII oocyte contains approximately 20000 to over800,000 mtDNA (4, 5); this range is 11000-428,000 in micefertilized oocytes and early embryos (6, 7). In addition, differences exist in the copies of mtDNA, ROS levels, and integrity of the cytoskeleton between in vitro matured (IVM) and naturally collected mice oocytes (8). 

Reactive oxygen species (ROS) is the byproduct of the oxidative phosphorylation chain system in mitochondria (9). Elevated levels of ROS cause oxidative stress and may lead to alterations in several redox pathways (10). The cells possess powerful enzymatic and non-enzymatic antioxidant defenses to protectagainst the damaging effects of ROS on DNA, lipids, and proteins (11). Excessive ROS or inadequate antioxidant protection within the cell results in oxidative stress. The cells can be protected against these effects by supplementation of culture media with antioxidants (12-14). Some antioxidants are synthesized by oocyte mitochondria (15). The exogenous antioxidant could improve oocyte and embryo development by decreasing ROS levels (13, 14, 16).

Selenium is a trace element present in the catalytic site of antioxidant enzymes such as glutathione peroxidase. In the form of sodium selenite (SS), it is used as a supplement in culture media and protects cells from oxidative damage (17). Abedelahi et al. (13, 16) have demonstrated that SS can improve the *in vitro* growth and maturation of mouse preantral follicles by reducing ROS levels and increasing the total antioxidant capacity and glutathione peroxidase activity of follicles. Similarly, Liu et al. (18) showed that SS significantly suppressed oxidative stress, by decreasing oxidative status of the cell and lipid peroxidation levels. Tareq et al. (19) reported an increase in the rate of maturation and embryo development in porcine oocytes cultured in the presence of selenium. The similar beneficial effect of SS in the culture media of porcine embryo was shown by a decrease in apoptosis and increase in expression of glutathione peroxidase (20). 

Little is known about mtDNA copy number changes during *in vitro* maturation of mouse oocytes in the presence of SS as an antioxidant. The present study determined the effects of SS supplementation of oocyte maturation medium on oocyte maturation, mtDNA copy number, and ROS levels in comparison with in vitro collected oocytes.

## Materials and Methods

Unless otherwise indicated, allchemicals were purchased from Sigma Aldrich (Germany). This experimental study used National Medical Research Institute (NMRI) female mice (n=48). The mice were housed in the Animal House at Tarbiat Modares University. The Ethical Committee of the Tarbiat Modares University approved this study (Ref No. 52.1637).

### Germinal vesicle oocyte collection

Adult female mice 6-8 weeks old (n=38) were superovulated by intraperitoneal injection (i.p.) of 10 IU pregnant mare serum gonadotropin (PMSG, Folligon, Intervet, Australia). Female mice were killed by cervical dislocation 48 hours after the PMSG injection and the dissected ovaries were placed in a-minimal essential medium (α-MEM, Gibco, UK) supplemented with 5% heat-inactivated fetal bovine serum (FBS, Gibco, UK). Antral follicles were punctured with needles to release the oocytes and the cumulus cells were mechanically removed. Oocytes that had a prominent germinal vesicle (GV) and clear ooplasm with 90 µm diameter were selected and collected (n=817).

Some of GV oocytes (n=778) were subjected to in vitro maturation in SS supplemented and non-supplemented groups. The other GV oocytes were analyzed by mitochondrial staining (n=10) and mtDNA copy number analysis (n=29).

### *In vivo* metaphase I and metaphase II oocyte collection

For harvesting the ovulated *in vivo* metaphase I (MI, OV-MI) and metaphase II (MII, OV-MII) oocytes, female mice (n=10) were superovulated by i.p. injection of 10 IU PMSG followed by another injection of 10 IU human chorionic gonadotropin (hCG, Sereno, Switzerland) 48 hours later. The oocytes were collected from the ampullary region of each oviduct 12-16 hours after the hCG injection. Cumulus cells were removed enzymatically by using 0.01% hyaluronidase. The oocytes with homogeneous and clear ooplasm that lacked any polar body or GV were considered OV-MI oocytes, those with one polar body were classified as OV-MII oocytes. The collected OV-MI were studied for mitochondrial staining using MitoTracker green (n=10) and for mtDNA copy number (n=15). These oocytes were individually stored at -80°C.

The OV-MII oocytes were analyzed for ROS concentration and mitochondrial staining using MitoTracker green (n=10) and for mtDNA copy number (n=15). These oocytes were individually stored at -80°C. 

### *In vitro* maturation

The GV oocytes were cultured in two groups, SS^+^ and SS^-^. The SS^+^ group (n=317 in 10 repeats) was cultured in α-MEM medium supplemented with 100 mIU/ml rFSH (Sereno, Switzerland), 10 IU/ml hCG, 10% FBS, and 10 ng/ml SS (13) under mineral oil at 37°C in 5% CO2 and air for 14 hours. The second group, or the media without SS supplementation (n=461 in 10 repeats), was considered to be the non-treated control group. After 14 hours, we morphologically assessed the oocyte maturation rate. Absence of GV within the ooplasm was used as the criteria for MI oocytes whereas extrusion of the first polar body was considered to be the criterion for MII oocytes. The matured MI and MII oocytes were classified as IVM-MI and IVMMII. These experiments were performed for at least 10 times and we assessed the collected oocytes as follows.

### Visualization of the mitochondria using MitoTracker green

The presence of viable mitochondria was identified by MitoTracker green (Molecular Probes, Invitrogen, Eugene, OR, USA) staining. We prepared a stock solution of MitoTracker green at a concentration of 1 mmol in DMSO and stored the solution at -20oC. The in vitro MII oocytes from both experimental groups and in vivo collected oocytes at the GV, MI, and MII stages (n=10 for each group and developmental stage) were stained with 0.2 mmol MitoTracker green in PBS at 37oC for 10 minutes. After washing in PBS, the oocytes were mounted on glass slides and observed under fluorescent microscope at the 490 wavelength (21). Then, a micrograph of each oocyte was prepared and imported into ImageJ software (National Institutes of Health, Bethesda, MD, USA). Next, we analyzed and compared the fluorescence intensity in different groups of oocytes.

### Reactive oxygen species analysis

The collected *in vitro* and in vivo MII oocytes were washed twice with PBS and incubated in 40 mmol/L of tris-HCl buffer (pH=7.0) that contained 5 mmol/L 2´,7´ dichlorodihydrofluorescein diacetate (DCF, Merck, Germany) at 37ºC for 30 minutes (n=60 for each group for three repeats of 20 pooled oocytes per repeat). Next, the oocytes were sonicated at 50W for 2 minutes, and centrifuged at 4°C and 10000 g for 20 minutes. Then, the supernatant was monitored using a spectrofluorometer at 488 nm excitation and 525 nm emission (22). Data were expressed as µM H_2_O_2_ and the mean of the DCF fluorescence intensity. A standard curve was prepared by fluorescence intensity of different concentrations of H_2_O_2_. 

### DNA extraction from individual oocytes 

We extracted DNA from completely denuded individual oocytes from all studied groups (n=15 for each developmental stage per group). A total of 10 µl of lysis solution that contained 50 mM tris-HCl (pH=8.5), 0.1 mM EDTA, 0.5% Tween-20, and 200 µg/ml proteinase K (Roche, Germany) were added to each tube followed by an overnight incubation at 55ºC. The samples were heated to 95ºC for 10 minutes to inactivate proteinase K. Each sample was used directly as template DNA for polymerase chain reaction (PCR).

### Primer design

We sought to identify the unique regions of the mouse mitochondrial genome with no pseudogene in the nuclear DNA. The entire sequence of mouse mitochondrial DNA was obtained from NCBI (NC_005089.1). The FASTA format of this sequence was split into 200 bp fragments with 50 bp overlaps. These fragments were searched against the mouse nuclear genome using NCBI Blast. The unique regions of the mitochondrial genome that had no duplicate in the nuclear genome were identified and used for primer design. Specific primers (Table 1) were design using Primer3Plus (http://sourceforge.net/projects/ primer3/) software and synthesized at MWG Germany.

**Table 1:** Mouse mitochondrial specific primer sequences


Primer code	Primer sequence (5´-3´)	Length (bp)

MTF	GCTAGTGTGAGTGATAGGGTAG	20
MTR	CCAATACGCCCTGTAACAAC	22


### Preparation of standard dilutions

In order to obtain standard curves we constructed standard DNA by cloning the PCR products. These products were amplified using the primer sets presented in Table 1 into the pTZ57R/T vector (Thermo Scientific Bio, USA). We used the MTF and MTR primers to amplify a 68 bp unique fragment of mtDNA. After electrophoresis, the amplified product was extracted from agarose gel by the ExpinTM Combo GP kit (GeneAll Biotechnology, Korea) according to the manufacturer’s protocol. The extracted product was cloned into the vector pTZ57R/T (Thermo Scientific, USA), purified, and sequenced. The recombinant plasmid was linearized and cleaned up by a GeneAll kit (General Biosystem, Korea). The product underwent spectrophotometry. The concentration of recombinant plasmid was calculated and diluted to 3×10^5^ copies/5 µl. We prepared four serial dilutions of standard DNA with at 1/10 standard concentration. These standard dilutions were kept at 4ºC until analysis and used in real- time PCR for mtDNA copy number quantification. 

### Quantification of mitochondria DNA copy number using real-time polymerase chain reaction

Real-time PCR was performed to determine the total amount of mtDNA of each single oocyte in all study groups. Each reaction contained 10 µl of SYBR green master mix (Applied Biosystems, USA), 2 µl primer mix (MTF and MTR), 3 µl of sterile water, and 5 µl of oocyte DNAextract (5 µl of each total DNAsample). Each oocyte DNA extract was divided into two wells as duplicates. All real-time runs included four concentrations of serial standard dilutions in triplicate (R^2^≥0.99). To rule out cross contamination a "no template control" (NTC) was added to each single real-time run. The reactions were performed with an ABI 7500 instrument (Applied Biosystems, CA, USA). Each PCR reaction included an initial denaturation step of 95ºC for 10 minutes, followed by 40 cycles of 95ºC for 15 seconds, 60ºC for 30 seconds, and 72ºC for 30 seconds. A melting curve stage was included at the end of the run to confirm the absence of non-specific products and primer dimerization. The copy number of mtDNA for each oocyte was calculated from both duplicate wells.

### Statistical analysis

Statistical analysis was performed using SPSS software (IBM SPSS statistics 22). All data were presented as mean ± SD. The normality of data was tested by the Kolmogorov-Smirnov test and the data of developmental rates of oocytes were compared by the t test. The mtDNA copy number and ROS level of oocytes were assessed by one-way ANOVA and Tukey’s HSD was used as the post hoc test. Statistical significance was P<0.05 for all analyses. 

## Results

### Maturation rate of oocytes

Table 2 summarizes the maturation rates of GV oocytes. The percent of oocytes which matured in the presence of SS were 6.85 ± 1.28 for the MI stage and 79.25 ± 0.52 for the MII stage. In the absence of SS, the maturation rate for GV oocytes was 6.36 ± 1.50 for the MI stage and 71.32 ± 3.78 for the MII stage. These rates were significantly higher in the SS supplemented group compared to the non-treated control group (P<0.05). 

**Table 2:** The maturation rates of GV oocytes in the presence and absence of sodium selenite


Group	Sodium selenite	Total number	Number of arrested GV (mean% ± SE)	Number of MI(mean% ± SE)	Number of MII(mean% ± SE)	Number of degenerated (mean% ± SE)

Control	-	461	73 (16.67 ± 2.14)	32 (6.36 ± 1.50)	335 (71.32 ± 3.78)	21 (5.63 ± 1.86)
Experiment	+	317	38 (11.97 ± 1.54)	22 (6.85 ± 1.28)	251 (79.25 ± 0.52)^*^	6 (1.90 ± 0.93)


* ; There was significant difference with the control group in the same column(P<0.05), GV; Germinal vesicle, MI; Metaphase I oocytes, and MII; Metaphase II oocytes.

### Mitochondrial distribution

**Fig.1:**
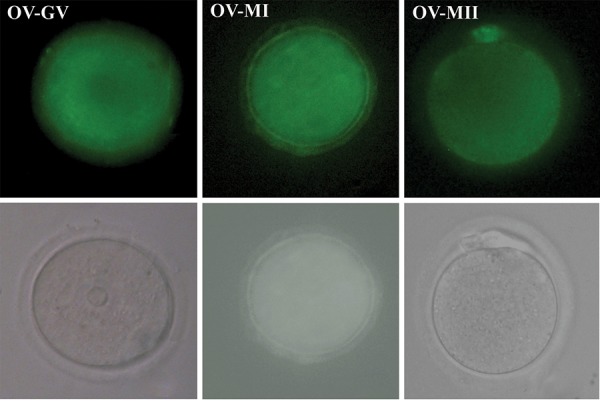
The oocytes at different developmental stages obtained from in vivo (OV) were stained for mitochondria by MitoTracker green. A. Germinal vesicle (GV), B. Metaphase I (MI), C. Metaphase II (MII) oocytes, and D-F. Phase contrast micrograph of the same group is shown in the second row. (scale bar: 30 µm).

**Fig.2:**
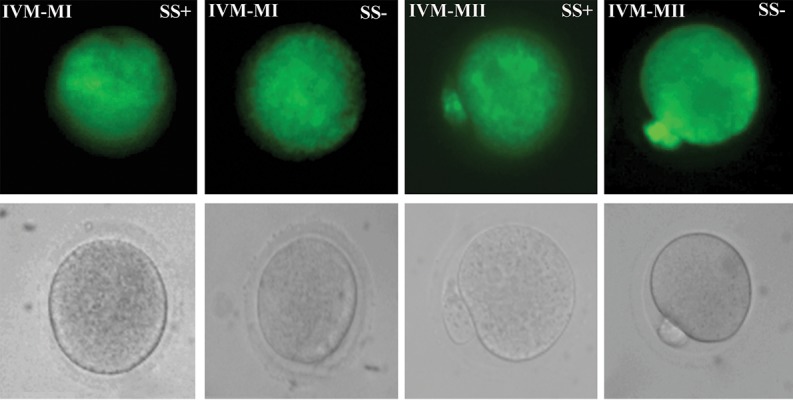
In vitro matured (IVM) oocytes at different developmental stages were stained for mitochondria by MitoTracker green. A. Metaphase I (MI) in the presence of sodium selenite (SS^+^), B. MI in the absence of sodium selenite (SS), C. Metaphase II (MII) in the presence of SS^+^, D. MII in the absence of sodium selenite (SS), and E-H. Phase contrast micrograph of the same group is shown in the second row (scale bar: 30 µm).

We observed mitochondrial distribution of oocytes at different developmental stages in the study groups with a fluorescent microscope using MitoTracker green. The representative micrographs of these oocytes were shown in Figure 1 and 2. The mitochondrial distribution in the cytoplasm of in vivo obtained oocytes consisted of a homogenously diffused pattern (Fig .1). There were some aggregations of mitochondria within the IVM oocytes (Fig .2). We observed similar patterns of mitochondrial distribution in all IVM oocytes with and without SS supplementation (Fig .2A-D). 

**Fig.3:**
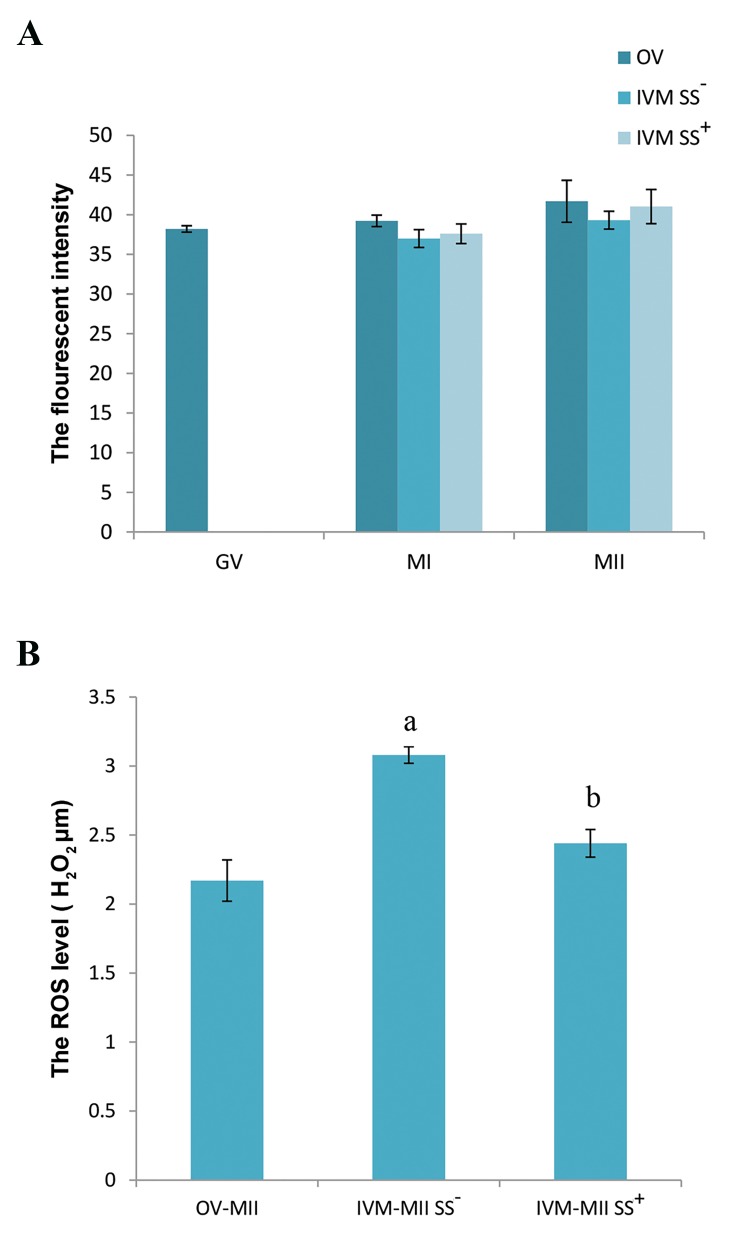
The relative fluorescence intensity and reactive oxygen species (ROS) levels in oocytes at different developmental stages. A. Relative fluorescence intensity with MitoTracker green staining in oocytes at different developmental stages obtained from in vivo (OV) and in vitro (IVM) conditions. There was no significant difference between groups. In the presence of sodium selenite (SS^+^) and in the absence of sodium selenite (SS) and B. ROS levels in MII oocytes derived from in vivo (control) and in vitro conditions. The minimum level of ROS was demonstrated in the in vivo obtained oocytes (OV-MII). There was a significantly lower ROS level in IVM oocytes in the presence of sodium selenite (SS^+^) compared to the non-sodium selenite SS^-^ treated group (P<0.05). GV; Germinal vesicle, MI; Metaphase I oocytes, and MII; Metaphase II oocytes.

The florescent intensities with regards to mitochondrial staining (Fig .3A) in GV oocytes was 38.21 ± 0.40. In the presence of SS, it was 37.62 ± 1.24 for IVM-MI and 41.02 ± 0.72 for IVM-MII. In the absence of SS, this finding was 36.99 ± 1.13 for IVM-MI and 39.32 ± 1.12 for IVMMII. Fluorescence intensity for OV-MI was 39.22 ± 0.72 and 41.69 ± 2.64 for OV-MII. There was no significant difference between the groups. 

### Reactive oxygen species concentration

The ROS levels in all studied MII oocytes (IVM and *in vivo* collected) were given in Figure 3. Data were shown as µM of H_2_O_2_. The level of ROS in IVM-MII oocytes in the presence of SS was 2.44 ± 0.10. In the group without SS, ROS was 3.08 ± 0.06 which was significantly lower in the SS supplemented group compared to the control group (P<0.05). The concentration of ROS in the OV-MII (2.17 ± 0.15) group was significantly lower than both IVM-MII oocyte groups (P<0.05).

### Mitochondrial DNA copy number

**Fig.4:**
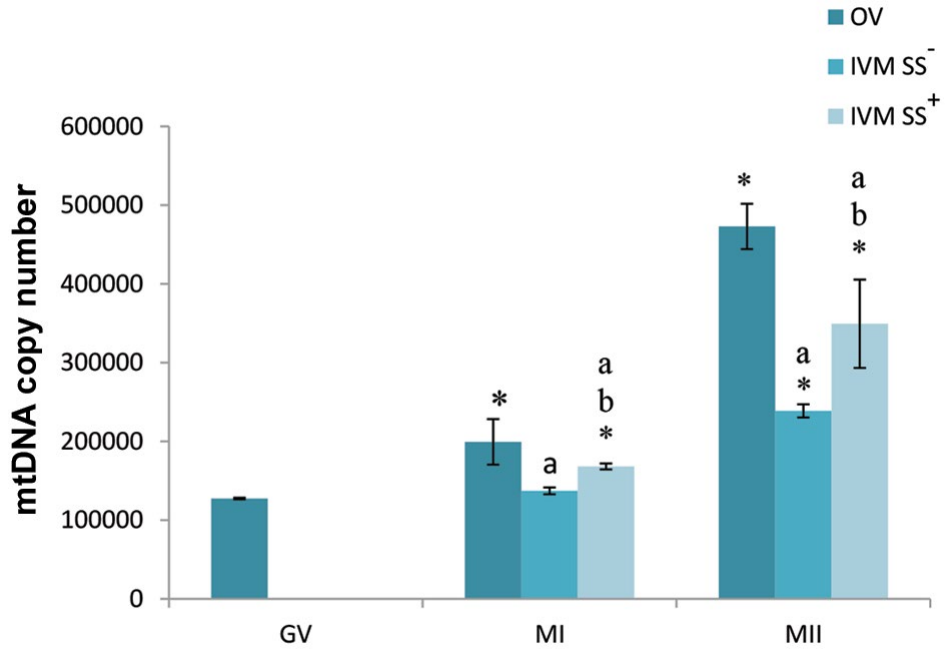
The absolute mitochondrial DNA (mtDNA) copy number of individual oocytes obtained from real-time PCR analysis. PCR; Polymerase chain reaction, OV; Oocytes obtained in vivo, IVM; Oocytes obtained from in vitro conditions, GV; Germinal vesicle, MI; Metaphase I oocytes, MII; Metaphase II oocytes, *; Significant differences with GV oocytes, a; Significant differences with OV oocytes in the same developmental stages, and b; Significant differences with IVM oocytes without (SS) in the same developmental stages.

The mean mtDNA copy number in single oocytes for all study groups is shown in Figure 4. This copy number in GV oocytes was 127,468.68 ± 1066.61. The copy number for OV-MI oocytes was 199,335.58 ± 28843.67, whereas for OV-MII oocytes it was 472,881.19 ± 28822.47. The IVMMI oocytes in the SS supplemented groups had a mtDNA copy number of 168,244.12 ± 3759.48. IVM-MII oocytes in the SS supplemented groups had a mean mtDNA copy number of 349,414.2 ± 56027.22. In the group without SS these numbers were 137,223.5 ± 4285.05 (IVM-MI) and 238,720.16 ± 8267.06 (IVM-MII). Oocytes from the SS supplemented group had a significantly higher mtDNA copy number compared to the group without SS (P<0.05). All IVM oocytes had significantly lower mtDNA copy numbers than their respected *in vivo* obtained oocytes (P<0.05). There was a significantly greater mtDNA copy number for all MII oocytes compared to both GV and MI oocytes in the same group (P<0.05). 

## Discussion

The present study, similar to other investigations, showed that the developmental competence of IVM oocytes was lower than *in vivo* obtained oocytes (5, 6). However, we demonstrated the beneficial effects of supplementation of culture media with SS as an antioxidant on oocyte maturation by reducing the ROS levels. A similar effect of SS on follicular development and oocyte maturation in mice and bovines has been previously shown by other investigators (13, 16, 23). Selenium acts via intracellular signaling factors that include protein kinase C, nuclear factor-kappa B, and inhibitors of apoptosis proteins (24, 25). Many of the biological actions of selenium are attributed to its powerful antioxidant properties, including direct quenching of ROS and chelation of metal ions (25). 

As our data demonstrated, all IVM-MII oocytes had higher ROS levels than *in vivo* obtained oocytes. This level in the SS treated group was lower than the non- treated group. Researchers previously reported increased ROS production during IVM of oocytes (23, 26). Our previous studies also showed that SS improved the in vitro development of follicles by increasing the follicular total antioxidant capacity level and decreasing the ROS level (13, 16). In agreement with this observation, Saito et al. (25) have reported that removal of selenium from the culture medium induced ROS production and cell death. Selenium suppresses oxidative stress by increasing the activity of antioxidant selenoenzymes and inhibits the activation of the PI3K/AKT and ERK signaling pathways stimulated by oxidative stress (18). 

An *in vivo* study by Said et al. (27) showed that SS had a radioprotective effect and improved rat folliculogenesis through increasing ovarian granulosa cell proliferation, and decreasing lipid peroxidation and oxidative stress. ROS is generated during ATP production within the mitochondria and its high level causes oxidative damage of mtDNA (28). On the other hand, mitochondria are especially sensitive to oxidative stress because of its minimal DNA repair enzymes compared to genomic DNA (29). It has been demonstrated that oxidative stress induces mtDNA degradation (28). Research has shown that DNA damage can interfere with POLRMT RNA primer synthesis and disrupt pol γ processivity and affect mtDNA replication (30). In this regard, Ge et al. (8) concluded that the non- physiological condition of controlled ovarian stimulation and *in vitro* maturation treatments inhibited mtDNA replication, altered mitochondrial function, and increased ROS production. Therefore, damage to the mitochondria might partly explain the low efficiency of assisted reproductive techniques and high rate of embryonic loss associated with these clinical procedures.

Overall, our results revealed a significantly lower mtDNA copy number for all IVM oocytes (MI and MII) compared to *in vivo* matured oocytes. This might explain that changes in mtDNA copy number could interfere with normal oocyte and embryo development. Therefore, IVM oocytes have lower potential for fertilization and further development. Ge et al. (8) also detected significant differences in the mtDNA copy number and level of ROS in mouse oocytes obtained from *in vitro* and *in vivo* conditions. 

Additionally, our data showed that mtDNAcopy number of oocytes increased significantly from the GV (127,468) to the MII (472,881) stages. The average mtDNA copy number determined in the present study was close to other investigations (6, 7). In contrast, no significant increase in mtDNA copy number from GV to IVM derived MII oocytes were reported in ovines and humans (4, 31). Attempts to quantify the amount of mtDNA in oocytes using PCR-based methods showed highly variable results. This discrepancy in mtDNA within the oocytes could by mainly related to technical error, different sources of oocytes (pooled or single), and different developmental stages of oocytes in several species (3-5, 32, 33).

Our results, for the first time, demonstrated that supplementation of maturation medium with SS could increase the mtDNA copy number of MI and MII oocytes compared to the non-treated group. Perhaps, the mitochondrial biogenesis in oocytes was stimulated during IVM in the presence of SS and was associated with higher developmental competence of the oocytes. In agreement with this suggestion, it has been shown that mtDNA copy number could change in response to environmental signals such as temperature, energy deprivation, nutrients, and growth factors (34).

This study showed that the mitochondrial distribution in IVM oocytes had some aggregation in comparison with in vivo obtained oocytes; however, the intensity of mitochondrial staining did not differ in these studied groups. Similarly, Stojkovic et al. (21) showed that the mitochondrial clumps became larger after IVM of oocytes. Liu et al. (35) demonstrated that the distribution of mitochondria in IVM oocytes differed slightly from that of in vivo obtained oocytes. They concluded that this different pattern resulted in the reduced developmental potential of IVM oocytes. Insufficient culture conditions might prevent mitochondrial migration within the ooplasm and affect cytoplasmic maturation (36). Thus, proper distribution of mitochondria during IVM of oocytes is critical for further development. In this regard, Kim et al. (14) have reported that treating oocytes with antioxidant could improve cytoplasmic maturation and cause morphologically uniform distribution of mitochondria and lipid droplets in the cytoplasm.

## Conclusion

SS increases oocyte mtDNAcopy number by decreasing oxidative stress and is associated with better oocyte developmental competence.
